# Whole-genome analysis of mumps virus genotype F in Shandong, China (2006–2018)

**DOI:** 10.3389/fmicb.2026.1912439

**Published:** 2026-09-09

**Authors:** Suting Wang, Ping Xiong, Yao Liu, Man Zhang, Yiping Feng, Xinyu Yuan, Xia Feng, Zexin Tao, Yingjie Zhang, Xiaolin Jiang

**Affiliations:** 1 Shandong Center for Disease Control and Prevention, Jinan, China; 2 Shandong Province Key Laboratory of Intelligent Monitoring, Early Warning, and Prevention of Infectious Diseases, Shandong, China

**Keywords:** genetic characteristics, incidence, mumps, vaccine strain, whole-genome

## Abstract

**Introduction:**

Despite the introduction of mumps-containing vaccines into the routine immunization program in 2008, mumps incidence in Shandong Province, China, declined but remained substantially higher than that of measles and rubella, and above WHO elimination thresholds, raising the possibility of whether genetic evolution of circulating strains has contributed to this phenomenon. To address, we compared mumps viruses isolated pre-vaccine (2006–2007) and a decade after rollout (2018).

**Methods:**

We analyzed mumps case data reported in Shandong from 2005 to 2024. We also performed whole-genome sequencing on 16 mumps virus strains isolated during the same period. Phylogenetic analyses were conducted to characterize viral genetic features and identify mutations.

**Results:**

From 2005 to 2024, 167,590 mumps cases were reported, peaking in 2012 (25.33 per 100,000 population). SH-gene typing consistently identified all 16 strains as genotype F, a result corroborated by whole-genome phylogeny; however, the SH gene alone proved insufficient for phylogenetic resolution among closely related strains, consistent with the value of whole-genome sequencing for epidemiological surveillance. The 16 full-length genomes shared 98.34–100% nucleotide identity. Phylogenetic analysis revealed intermingling of 2006–2007 and 2018 isolates rather than time-specific clustering, with low inter-group p-distances. Importantly, between the two temporal groups, no significant differences were detected in their respective identities to the vaccine strain S79 at the whole-genome level (nucleotide or amino acid). Two positively selected sites were identified across the seven structural proteins (posterior probability > 0.95). Eight mutations in HN and F that alter hydrophilicity, hydrophobicity, or charge were found, but structural modeling predicted no appreciable conformational changes.

**Conclusion:**

We provides a whole-genome-based, two-time point comparison of genotype F mumps viruses in Shandong across a ~ 12-year interval spanning vaccine introduction (2006–2007 vs. 2018). Overall, the Shandong 2018 isolates remained as similar to the 2006–2007 isolates as F strains are nationwide, and both groups were comparably close to vaccine strain S79, indicating no detectable genetic divergence or drift over the ~12-year period. As the SH gene alone lacked sufficient diversity to resolve closely related isolates, whole-genome sequencing provided more accurate genetic characterization in this study.

## Introduction

Mumps is a prevalent respiratory infectious disease caused by the mumps virus (MuV) and usually manifests as swollen salivary glands, fever, and pain (Chen et al., 2024; Hviid et al., 2008). MuV belongs to the order Mononegavirales, the family Paramyxoviridae and the genus Rubulavirus. Its genome is a nonsegmented, single-stranded, negative sense RNA. It encodes seven structural proteins, which are nucleocapsid (N), phospho (P), matrix (M), fusion (F), small hydrophobic (SH), hemagglutinin-neuraminidase (HN), and large (L) proteins (Kim et al., 2007). Currently, 12 genotypes (A–N, excluding E and M) are recognized based on sequence analysis of the entire 316-nucleotide SH gene (Barrabeig et al., 2007). HN protein is the main target of neutralizing antibodies (Deng et al., 2012; Šantak et al., 2013). Genotype F is predominant and indigenous in China mainland (Cui et al., 2017; Cui et al., 2013; Jin et al., 2015).

Before the application of mumps-containing vaccines (MuCVs) in the late 1960s, mumps caused widespread morbidity (Hviid et al., 2008; Russell et al., 2018). The live-attenuated vaccine for mumps and subsequent combination measles, mumps, and rubella vaccine (MMR) greatly reduced the incidence of mumps worldwide (Di Pietrantonj et al., 2021; Hilleman et al., 1968). But mumps outbreaks are still common in the world (Echevarría et al., 2010; Santos et al., 2006). The reasons for this are not yet fully elucidated but contributing factors may include waning immunity, variability in antibody responses, and antigenic differences between the vaccine and circulating wild-type strains (Santos et al., 2006; Gokhale et al., 2023; Latner et al., 2017; Lewnard and Grad, 2018; Rubin et al., 2008; Yang et al., 2010). Previous studies have documented genetic differences between genotype F field strains and the genotype A vaccine strain S79 in China; however, available evidence indicates that vaccine-induced antibodies retain cross-neutralizing activity against genotype F viruses (Cui et al., 2018). Nevertheless, whether circulating genotype F viruses in Shandong have undergone additional genetic divergence relative to the vaccine strain over the extended period following vaccine introduction has not been systematically assessed at the whole-genome level.

Mumps-containing vaccines (MuCV) were incorporated into Shandong Province’s Immunization Program in 2008 (Xu et al., 2018). Since then, a single dose of (MuCV) has been administered to children at 18 months of age. However, according to the national mumps Surveillance System in China (Shandong Provincial Center for Disease Control and Prevention, unpublished data), the mumps morbidity remained high until 2014 when it dropped to 8.31 cases per 100,000 population following the introduction of a 2-dose Measles-Mumps-Rubella Vaccine (MMR) schedule administered at ages 18 months and 6 years. During the COVID-19 pandemic, the vaccination schedule was adjusted to 2 doses of MMR given at ages 8 and 18 months, which further reduced the incidence rate to 2.86 cases per 100,000 population, but was still high for vaccine-preventable disease, such as measles and rubella. To assess whether circulating genotype F mumps viruses in Shandong have undergone detectable genetic divergence over the ~12-year period surrounding vaccine introduction, we compared whole-genome sequences of isolates from 2006–2007 (pre-vaccine) and 2018 (ten years post-introduction).

## Materials and methods

### Specimen collection and virus isolation

All the information of mumps cases was obtained from national mumps Surveillance System in China. Throat swabs were collected from patients with the symptoms meeting mumps diagnosis criteria within seven days after an onset. Patients’ demographic information was enrolled at the same time. The swabs were transported to Shandong Provincial Center for Disease Control and Prevention Measles/Rubella Net Laboratory for isolation and identification of MuV. MuV was isolated in Vero/hSLAM cells and harvested when CPE reached 75–90%. Full-length SH gene was amplified by one-step reverse transcription polymerase chain reaction (TaKaRa), and the purified products were Sanger-sequenced using a BigDye Terminator v3.1 kit on an ABI 3100 Genetic Analyzer (Cui et al., 2017). From 2006 to 2024, a total of 16 mumps strain was isolated, including two time windows (2006–2007 VS 2018), which were isolated before and after the MuCV was incorporated into Shandong Province’s Immunization Program in 2008.

### Joinpoint regression

The Joinpoint regression was performed using version 6.1.0 with a log-linear model. The maximum number of joinpoints was set to 3, and the optimal model was selected by minimizing the Bayesian Information Criterion (BIC). Error structures were assumed uncorrelated, and heteroscedasticity was accounted for by weighting based on the variance of the observed rates, assuming a Poisson distribution. The annual percent change (APC) for each segment was calculated, and its statistical significance was assessed by t-test at *α* = 0.05. These details have now been added to the Methods section.

### RNA extraction, library preparation and sequencing

Viral nucleic acid was extracted from the viral isolation using MagMAX Pathogen RNA/DNA Kit (Thermo Fisher, Lithuania). Library preparation was performed using Revelo™ RNA-Seq High Sensitivity library preparation kit (Tecan, Switzerland). After DNA removal and rRNA reverse transcription blocking, first-strand complementary DNA (cDNA) is generated, followed by a second-strand cDNA synthesis. Next, cDNA is subjected to Single Primer Isothermal Amplification. After cDNA end repairment, a single ‘A’ nucleotide is added to the 3′ ends of the blunt fragments. Then the adaptor is configured to ligate adaptors with the cDNAs. The PCR reaction system and program are configured and set up to amplify the cDNAs. After denaturation, single-stranded cDNAs are subsequently cyclized. Single-stranded circle DNA molecules are replicated via rolling cycle amplification, and a DNA nanoball (DNB) is generated. Sufficient quality DNBs are then loaded into patterned nanoarrays using high-intensity DNA nanochip technique and sequenced through combinatorial Probe-Anchor Synthesis (cPAS).

After sequencing, clean reads were obtained by removing reads containing adapters, reads shorter than 150 bp, reads containing poly-N, and low-quality reads (more than 30% of bases with Q-values below 20). Read quality values were encoded using Phred+33 For each sample, raw reads ranged from 33.94 to 70.07 million, and clean reads ranged from 33.28 to 67.45 million, with Q20 values ranging from 96.24 to 97.25% and Q30 values ranging from 92.10 to 93.24% across all samples. We performed both *de novo* and reference-guided assembly strategies. *De Novo* assembly of the clean data was conducted in CLC Genomics Workbench 12.0 (CLC Bio, Qiagen, Hilden, Germany) using the default options. Contigs > 200 bp in length and with > 10 × coverage was queried for sequence similarity using Basic Local Alignment Search Tool (BLAST) (https://blast.ncbi.nlm.nih.gov/Blast.cgi). BLASTN search against the NCBI viral reference database confirmed the presence of mumps virus reads as the predominant species in all samples. An additional resequencing assembly of the clean reads was carried out to obtain whole-genome of mumps. Following de novo assembly, A reference-guided assembly of the clean reads was carried out against the genotype F reference genome (GenBank accession No. KF170916.1) to recover the complete genome sequences. The assembled genome lengths ranged from 15,341 to 15,349 bp, with GC contents ranging from 42.47 to 42.64%, and alignment against the reference genome confirmed that >99% of the genome was covered for all 16 isolates, indicating near-complete genome recovery. The median read coverage depth across the 16 genomes ranged from approximately 100 × to 300×, comparable to or exceeding levels reported in other mumps whole-genome studies. Genome completeness was further verified by alignment against the reference genotype F genome (GenBank accession No. KF170916.1).

### Homologous comparison and phylogenetic analysis

Multiple sequence alignment, key site identification and sequence similarities were performed by the BioEdit software (version 7.0.5.3). To compare the genetic conservation relative to the vaccine strain S79 across the two temporal windows, we extracted the pairwise nucleotide and amino acid identity percentages for each structural protein (HN, F, M, N, SH, L and V/P) from sequence alignments. Between-group differences (2006–2007 vs. 2018) were evaluated using the two-sided Mann–Whitney U test, which does not assume normal distribution and is appropriate for small sample sizes. For the Mann–Whitney U tests across the seven structural proteins at both nucleotide and amino acid levels (14 tests in total), *p*-values were adjusted using the Benjamini–Hochberg procedure to control the false discovery rate at q = 0.05. All statistical analyses were conducted using R version 4.3.2 Phylogenetic analysis was performed using MEGA 11.0 software with the neighbor-joining method based on the Kimura 2-parameter model and 1,000 bootstrap replicates.

### Selective pressure

Site-specific selection analysis was conducted to estimate nonsynonymous (dN) and synonymous (dS) substitution rates. Seven codon substitution models were compared to identify positively selected sites (*ω* > 1, where *ω* = dN/dS), including: M0 (one-ratio) vs. M3 (discrete), M1a (nearly neutral) vs. M2a (positive selection), M7 (*β*) vs. M8 (*β* + *ω* > 1), and M8a (*β* + *ω* = 1) vs. M8. The M7 vs. M8 comparison served as the most stringent test for positive selection. Model comparisons were evaluated using likelihood ratio tests (LRTs) with a significance threshold of *p* < 0.05. Additionally, an empirical Bayes approach was employed to predict codon sites under positive selection, with posterior probabilities >0.95 considered strong evidence of reliable detection. All analyses were performed using the CODEML program implemented in EasyCodeML v1.21. The following parameter settings were applied: codon frequency model = F3 × 4; initial *ω* values were set to 0.4 for M0 and M1a, and 1.0 for M2a, M7, M8, and M8a; the *κ* and *ω* parameters were optimized during likelihood iterations; the NSsites parameter was set to 0, 1, 2, 3, 7, 8, and 10 for the respective models; and the number of rate classes for M3 and M8 was set to 3 and 11, respectively. Each model was run independently three times with different initial ω values to avoid convergence to local optima. For the likelihood ratio tests (LRTs) across the seven structural proteins, we adjusted the raw *p*-values using the Benjamini–Hochberg (BH) procedure to control the false discovery rate (FDR) at *q* = 0.05. A corrected *q*-value < 0.05 was considered statistically significant for positive selection.

### Structural modeling and mutagenesis analysis

The three-dimensional structures of the F and SH proteins were generated using SWISS-MODEL (https://swissmodel.expasy.org) based on the template structures with PDB IDs 9EXA (for the F protein) and 9DRQ (for the SH protein). The predicted model was visualized and analyzed using PyMOL (Version 2.5.0, Schrödinger, LLC). Model quality was assessed using the Global Model Quality Estimation (GMQE) and QMEANDisCo scoring functions. According to the SWISS-MODEL evaluation, both models exhibited GMQE scores above 0.40 and QMEANDisCo Global scores exceeding 0.50, indicating moderate to acceptable model quality, which is consistent with the moderate sequence identity between the target proteins and the available templates.

Site-directed mutagenesis (M20L and I151V) was performed computationally using the Mutagenesis Wizard implemented in PyMOL. Briefly, the wild-type structure was loaded into PyMOL, and duplicates were created for the mutant constructs. For each mutation, the target residue was selected and replaced with the desired amino acid using the rotamer library available in PyMOL. The optimal rotamer was selected based on the lowest steric strain energy to ensure minimal steric clashes with the surrounding environment. The backbone root-mean-square deviation (RMSD) between wild-type and mutant structures was calculated to assess structural conservation.

Molecular surfaces were generated using the Surface functionality in PyMOL with a probe radius of 1.4 Å. Mutated residues were highlighted as red spheres for visual identification. Structural representations were rendered at 300 dpi resolution for publication-quality figures.

## Results

### Mumps incidence in Shandong Province

**Figure 1:**
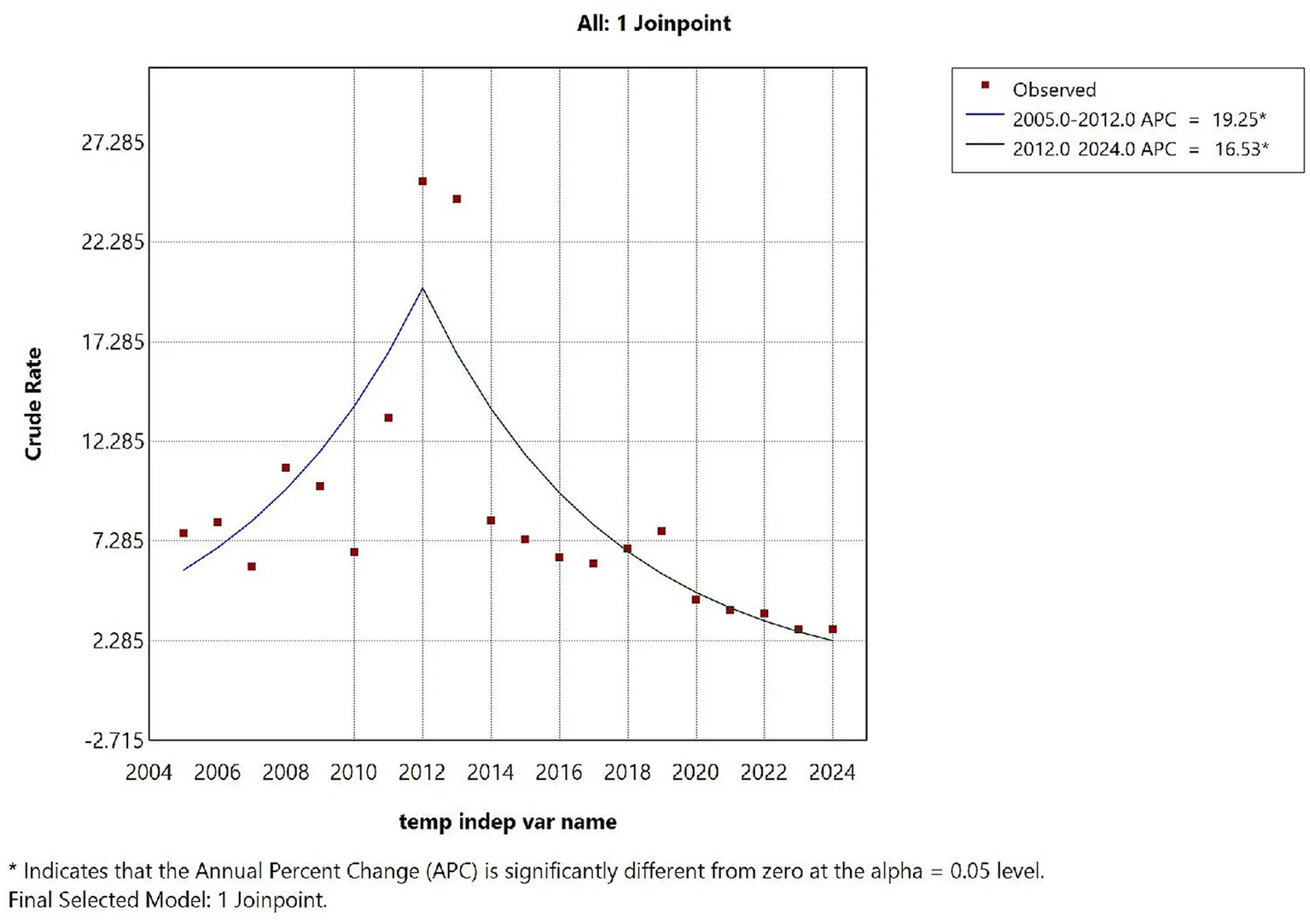
Annual mumps incidence in Shandong, 2005–2024, with Joinpoint regression trend lines. Incidence increased significantly from 2005 to 2012 (APC = 19.25%, *p* < 0.05) and decreased significantly from 2012 to 2024 (APC = −16.53%, *p* < 0.05). APC, annual percent change. Rates are per 100,000 population.

The annual population of Shandong Province from 2005 to 2024 ranged from approximately 91.98 million to 101.49 million, among them, a total of 167,590 mumps cases were reported, with an average annual incidence rate of 8.60 per 100,000 population. The peak incidence rate reached 25.33 per 100,000 population in 2012. Joinpoint regression analysis was applied to mumps morbidity data from 2005 to 2024 to examine temporal trends. The optimal model identified a single joinpoint in 2012 (95% CI, 2010–2014), with BIC-selected model. No other joinpoints (including 2008, 2014, or 2020) were selected as the optimal model. The analysis revealed a statistically significant difference in morbidity between the periods 2005–2012 and 2012–2024 (*p* = 0.001). Joinpoint regression analysis revealed two distinct trends: a significant increase from 2005 to 2012 (APC = 19.25, 95% CI: 8.53–49.16, *p* = 0.004), followed by a significant decrease from 2012 to 2024 (APC = −16.53, 95% CI: −26.21 to −12.54, *p* < 0.001) (Figure 1).

### Basic information and genetic analysis of Shandong mumps strains by complete genomic sequences

We recovered 16 complete mumps virus genomes from clinical specimens collected in Shandong between 2005 and 2024, representing two distinct temporal clusters: seven strains from 2006, one from 2007, and eight from 2018. Among these cases, 10 were male and 6 were female. The majority (75%, *n* = 12) were under 15 years of age, while 2 cases (12.5%) were between 15 and 20 years old, and another 2 (12.5%) were over 20 years old. Only one individual had received one dose of a mumps-containing vaccine (MuCV).

**Figure 2:**
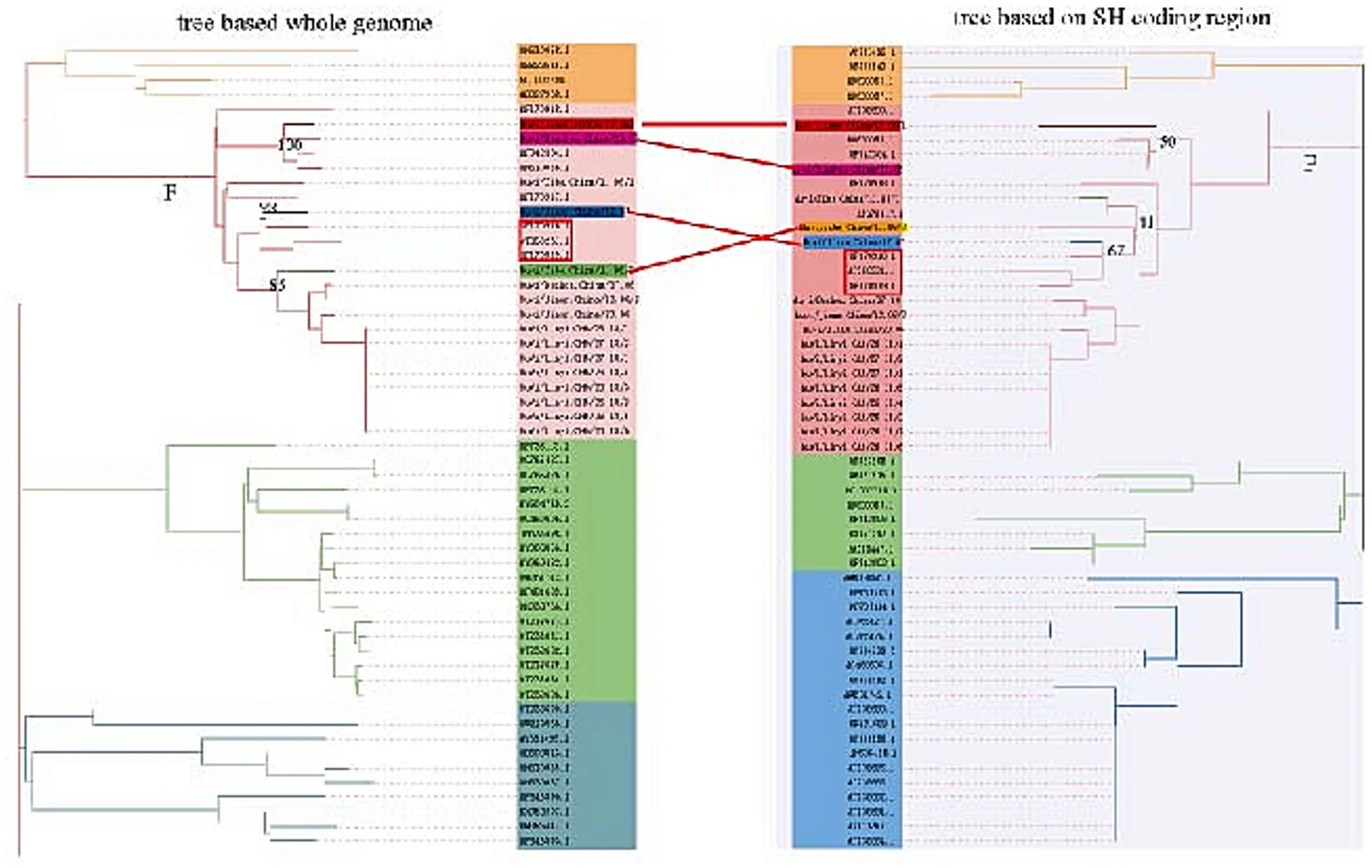
Phylogenetic trees of 16 Shandong isolates based on complete genomes **(A)** and the SH gene **(B)**. Both trees place all strains within genotype F, with isolates from 2006–2007 and 2018 intermingled. Trees were constructed using the neighbor-joining method with 1,000 bootstrap replicates.

To determine the genotype of the Shandong strains, a phylogenetic tree was constructed based on the 316-nt fragment of the SH gene, using 16 local isolates and 40 reference sequences. The analysis confirmed that all strains belonged to genotype F. Whole-genome sequencing of the 16 local isolates was subsequently performed, and the resulting sequences, together with the 40 references, were used to further validate the clustering of these genotype F isolates. Phylogenetic analysis based on complete genomic sequences yielded results largely consistent with those from the 316-nt SH gene fragment. All Shandong strains clustered together with reference sequences of genotype F in trees generated from both genomic regions. However, minor branching discrepancies were observed for four strains (MuVi/Jinan. China/12.06/1, MuVi/Binzhou. China/23.06, MuVi/Jinan. China/12.07, and MuVi/Zibo. China/11.06/2) at the terminal nodes. Notably, MuVi/Zibo. China/11.06/2 occupied different phylogenetic positions relative to reference sequences KF170916.1, MT350231.1, and KF170919.1 across the two trees. Due to limited variable sites in the SH gene, the SH-gene tree exhibits lower bootstrap support (<70%) at terminal nodes; however, the whole-genome tree shows high bootstrap support (>90%) at these branches. Isolates from 2006–2007 and 2018 were interspersed across the tree rather than forming distinct time-specific clades, indicating no clear temporal clustering at the resolution of the current dataset (Figure 2).

**Figure 3:**
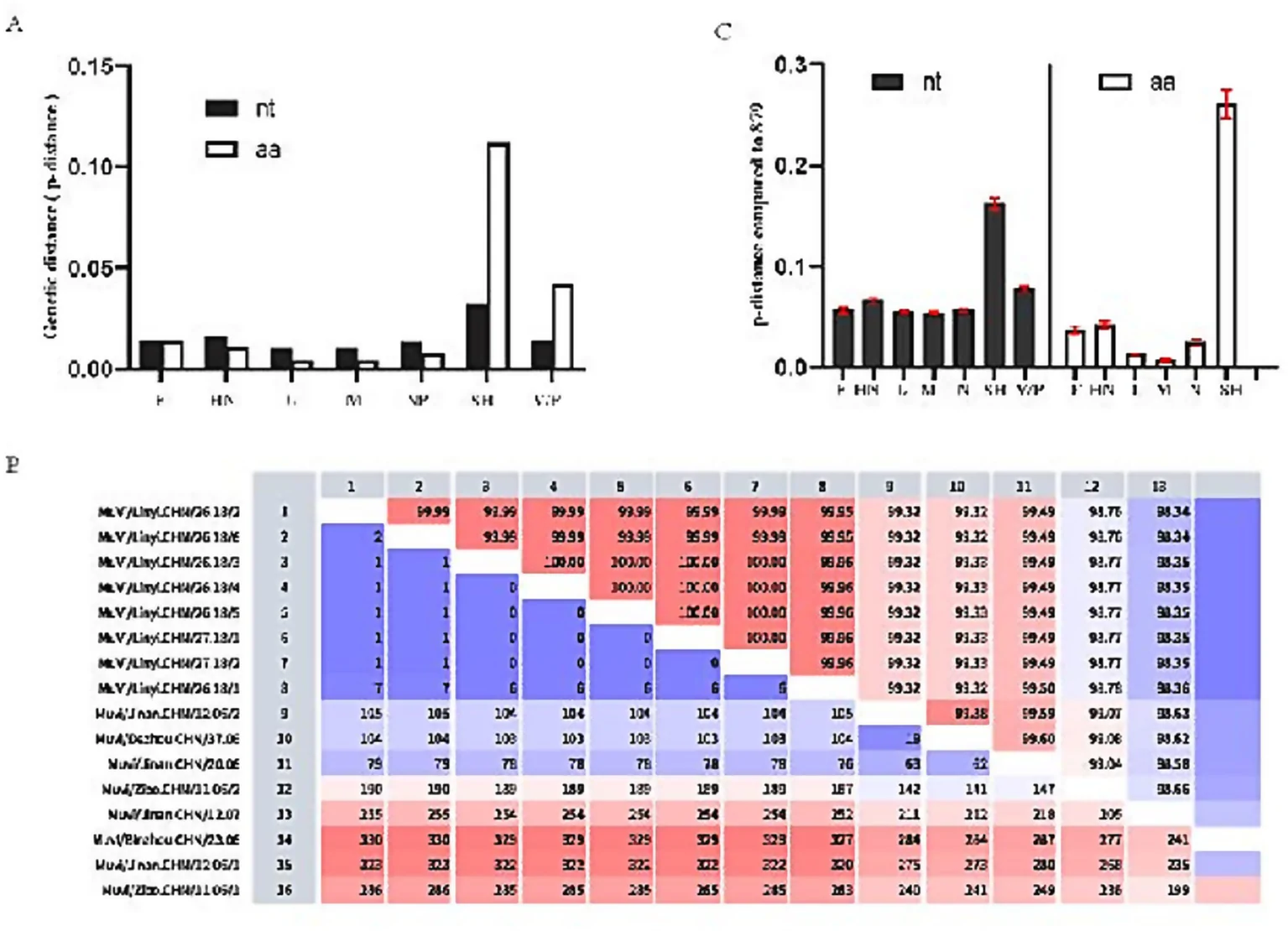
Homology analysis of Shandong mumps strains. **(A)** Average p-distances of the seven coding regions at the nucleotide (nt) and amino acid (aa) levels. **(B)** Pairwise nucleotide identities and difference counts across complete genome sequences. **(C)** P-distances between Shandong strains and the vaccine strain S79 at the nucleotide and amino acid levels.

Diversity across seven distinct regions of the whole genome was analyzed. Nucleotide diversity was highest in the region encoding the SH protein (overall mean *p*-distance = 0.03), while the regions encoding L and M were the most conserved (overall mean p-distance = 0.01). Amino acid diversity followed a similar pattern: proteins SH and V/P exhibited the highest divergence, whereas proteins L and M remained the most conserved. Notably, the SH protein showed greater genetic distance (overall mean p-distance = 0.112) than its corresponding nucleic acid region. In contrast, the highly conserved L and M proteins displayed even lower variability than their encoding nucleotides (Figure 3A). The SH gene is the only gene for which the amino acid p-distance is substantially higher than the nucleotide p-distance, indicating that the SH gene has the highest proportion of non-synonymous mutations—a finding fully consistent with the known high variability of the SH protein. In contrast, for the other six genes, the amino acid p-distances are lower than or comparable to the nucleotide p-distances, suggesting that the differences in these genes are predominantly synonymous substitutions under purifying selection. Additionally, we computed the identity and difference counts across the complete genomic sequences, including non-coding regions. The results revealed nucleotide identities ranging from 98.34 to 100%, with the number of differences varying from 0 to 330 nt (Figure 3B).

To assess genetic divergence between the two temporal clusters, we calculated the mean p-distances between the 2006–2007 group (*n* = 8) and the 2018 group (*n* = 8) for each gene. At the nucleotide level, the inter-group distances were 0.016 for *f*, 0.016 for *hn*, 0.010 for *l*, 0.009 for *m*, 0.005 for *n*, and 0.014 for *v/p*. At the protein level, the corresponding distances were 0.013 (F), 0.013 (HN), 0.030 (L), 0.004 (M), 0.003 (N), and 0.024 (V/P) (Table 1).

**Table 1:** Mean p-distances between the 2006–2007 group (*n* = 8) and the 2018 group (*n* = 8) at the nucleotide and protein levels.

Gene	Nucleotide p-distance	Protein p-distance
*f*	0.016	0.013
*hn*	0.016	0.013
*l*	0.01	0.03
*m*	0.009	0.004
*n*	0.005	0.003
*v/p*	0.014	0.024

Positive selection sites were identified using the CODEML algorithm implemented in EasyCodeML 1.21. Analysis under seven evolutionary models revealed that the M8 model provided a significantly better fit than M7 for F, HN, SH, and NP (BH-adjusted *q* < 0.05; Table 2). Under the M8 model, two sites under positive selection with a posterior probability exceeding 0.95 under the M8 model (*β* + ωs > 1): site 34 (H) in SH protein and site 468 (F) in the NP protein. An additional 7 sites with BEB probabilities between 0.90 and 0.95 are listed in Table 2 as tentative candidates for future validation.

**Table 2:** Parameter estimates, likelihood ratio test (LRT) results, and Benjamini–Hochberg (BH) adjusted *Q*-values for positive selection analysis across the seven structural proteins of mumps virus.

Structural protein	Model	Estimates of parameters	LRT *P*-value	BH-adjusted *Q*-value	Positively selected sites
F	M8	*p*1 = 0.00186, *ω* = 65.22869	2.20E-08	5.77 E-08	151 I 0.913
HN	M8	*p*1 = 0.00199, *ω* = 31.60141	1.01E-07	1.77 E-07	6 L 0.938, 120 A 0.914, 473 T 0.905
SH	M8	*p*1 = 0.30112, *ω* = 10.04465	5.46E-05	1.00E+00	20 M 0.941, 29 I 0.911, 34 H 0.955*
NP	M8	*p*1 = 0.00183, *ω* = 89.50219	1.00E-09	1.00E+00	453 N 0.924, 468 F 0.953*
L	M8	*p*1 = 0.00001, *ω* = 1.00000	9.99E-01	7.18 E-05	-
M	M8	*p*1 = 0.00267, *ω* = 83.87729	9.99E-01	5.25E-09	-

### Comparison analysis of Shandong strains and vaccine strains (S79)

The nucleotide sequence identity between Shandong strains and the vaccine strain S79 (genotype A) was lowest in the *sh* coding region, ranging from 82.2 to 84%. Similarly, the lowest amino acid identity was also observed in the SH protein, varying between 71.9 and 75.4%. The other six coding regions and their corresponding proteins showed high identity levels, all exceeding 90%. To further evaluate genetic divergence, p-distances between S79 and Shandong strains were calculated. The mean nucleotide p-distance across the seven coding regions ranged from 0.05 to 0.20, while the mean amino acid p-distance ranged from 0.01 to 0.30. Consistent with the identity results, the SH region exhibited the highest p-distance. In contrast, the HN protein—a major target of neutralizing antibodies—showed a conserved profile, with p-distances ranging from 0.038 to 0.048, amino identity ranging from 95.1 to 99.8%. For another important target of neutralizing antibodies(F protein), compared to S79, the p-distances ranged from 0.031 to 0.040 and amino acid identity ranged from 96.2 to 99.4% (Figure 3C).

**Figure 4:**
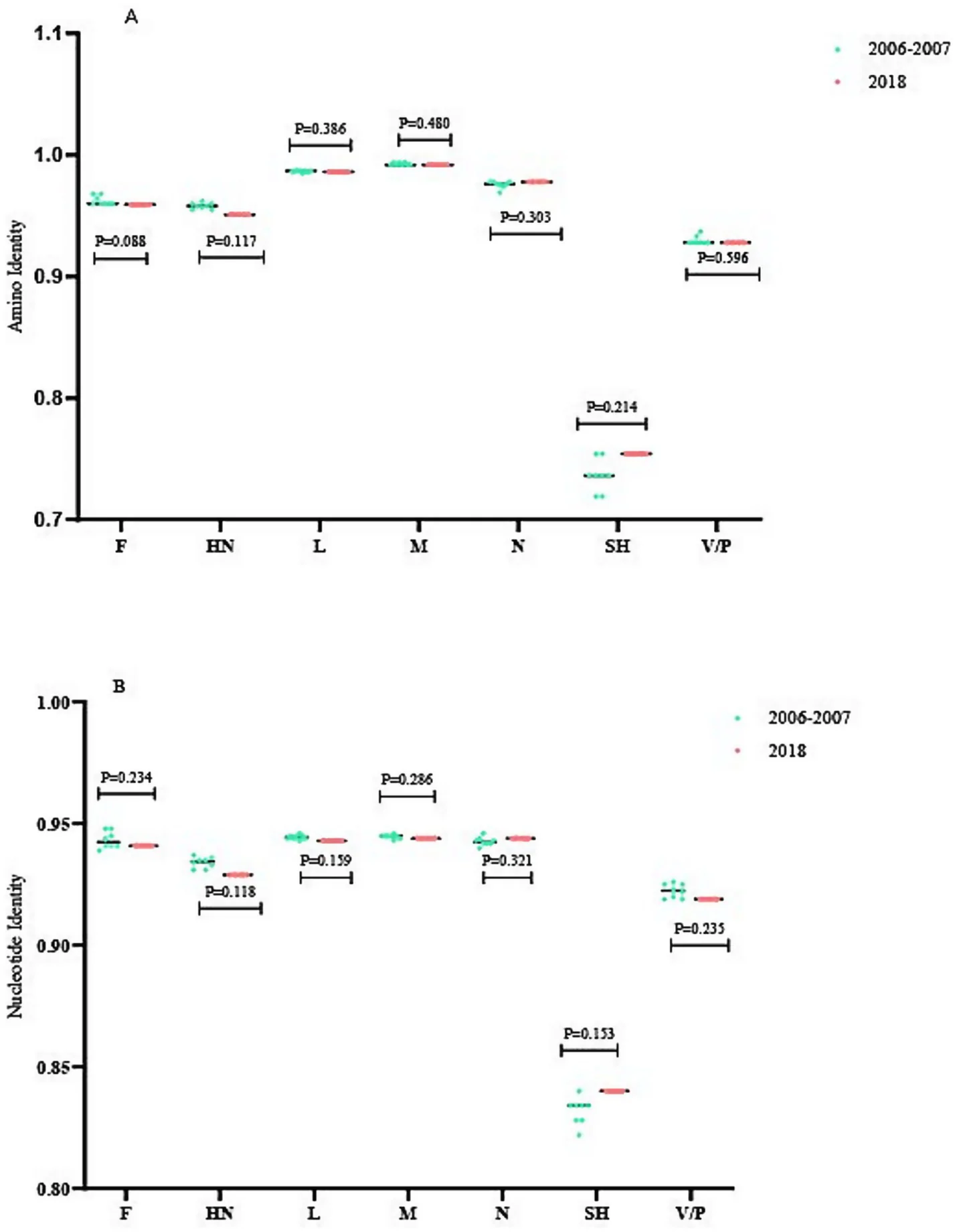
Nucleotide and amino acid identities of the seven structural proteins (F, HN, L, M, N, SH, and V/P) between Shandong isolates and the vaccine strain S79, compared between the 2006–2007 (*n* = 8) and 2018 (*n* = 8) groups. The SH protein showed the lowest identity in both groups. No significant differences were observed between the two time windows for any protein (Mann–Whitney U test, all *p* > 0.05). *p*-values from Mann–Whitney U tests are shown above the horizontal lines in the figure.

We next assessed whether the degree of sequence conservation relative to the vaccine strain S79 differed between the 2006–2007 and 2018 isolates. That is, for each group, we calculated the identity of each isolate relative to S79, and then compared these identity values between the two groups using Mann–Whitney U tests. The median nucleotide and amino acid identities for all seven structural proteins (HN, F, M, N, L, SH and V/P) were all high than 90%, except SH, in both temporal groups. Formal statistical comparison using the Mann–Whitney U test revealed no significant differences between the two groups in their respective genetic identities to S79 for any of the proteins examined (*p* > 0.05) (Figure 4). The Benjamini–Hochberg (BH) procedure was performed to control the false discovery rate (FDR) at *q* = 0.05. After BH correction, only the test for the F protein at the amino acid level yielded an adjusted *p*-value slightly below 0.05 (0.00450). All other comparisons were non-significant after correction.

We further compared key amino acid variations between the Shandong strains and the vaccine strain S79. A systematic screening of all 29 glycosylation sites across the seven structural proteins revealed only one alteration: strain MuVi/Linyi. CHN/26.18/1 exhibited an N12D mutation at the 12th–14th glycosylation site in HN protein. As this mutation was observed in only a single isolate, it likely represents a transient variant rather than a fixed polymorphism; functional implications remain speculative without experimental validation. Because F and HN are the main targets of neutralizing antibodies and are most relevant to vaccine effectiveness, we performed the analysis of hydrophilicity/hydrophobicity and charge physicochemical changes on the F and HN proteins. Several mutations affecting hydrophilicity/hydrophobicity or charge were identified in the F protein. These include S92P and S97L, located in solvent-exposed flanking regions near the proteolytic cleavage core motif; S16Y and S15P within the signal peptide region (sites 1–19); S409A in the head–neck region (sites 369–425) of the ectodomain; and I500T in the transmembrane segment (sites 483–519). Although multiple mutations were also present in the HN protein, only two were predicted to alter hydrophilicity/hydrophobicity or charge: V287I and I288K/T, both located within the major antigenic site (residues 329–420).

**Figure 5:**
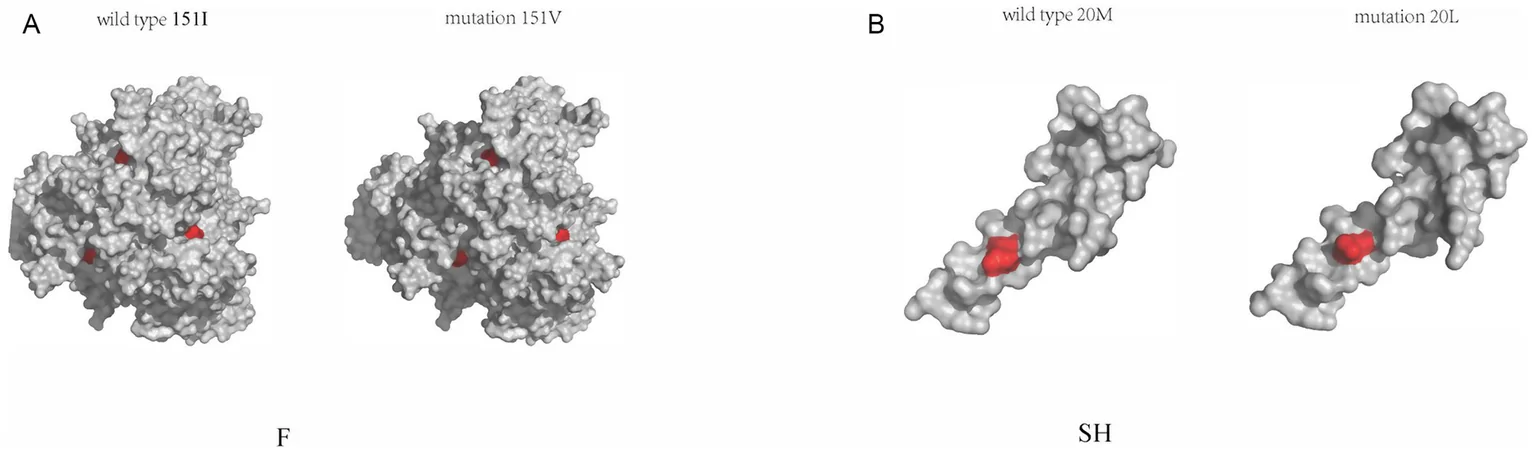
Wild-type and mutant structures of the two positively selected sites: **(A)** SH M20L and **(B)** F I151V. Mutated residues are highlighted in red. RMSD values (0.1 Å and <0.1 Å, respectively) confirm structural conservation, with both values below the 0.5 Å threshold. Structures were modeled using SWISS-MODEL and visualized with PyMOL. Structural modeling was restricted to sites that met three criteria: (i) posterior probability > 0.90, (ii) coverage by available template structures in SWISS-MODEL, and (iii) amino acid difference from the vaccine strain.

Because no structural model covering the N12D glycosylation site was available, we restricted our protein conformation analysis to positively selected sites meeting the following criteria: posterior probabilities > 0.90, coverage by the available structural models, and amino acid differences relative to the vaccine strain. This yielded two candidate sites: M20L in the SH protein and I151V in the F protein, both of which are located within *β*-sheet regions. Following the M → L substitution at position 20 of SH, the root-mean-square deviation (RMSD) was 0.055 Å, and following the I → V substitution at position 151 of F, the RMSD was 0.035 Å. Both values are well below 0.5 Å, indicating that these mutations do not disrupt the main-chain backbone architecture and that the overall protein structures remain stable. The schematic diagrams before and after mutation at the two sites are shown in Figure 5, with the targeted sites highlighted in red.

## Discussions

Mumps-containing vaccines (MuCV) were first incorporated into Shandong Province’s Immunization Program in 2008 as a single-dose regimen for children. But the incidence of mumps continued to rise until a turning point was observed in 2012. This coincided with a national mumps epidemic and reflected the limited effectiveness of the single-dose vaccine strategy used before 2014, rather than a decline in vaccine coverage or a change in viral characteristics. Similar patterns have been reported in other Chinese provinces (Xu et al., 2018; Su et al., 2016; Wang et al., 2012; Deng et al., 2012). This result supported the suggestions that second dose of the MMR vaccination should be highly recommended to prevent genotype F MuV wild-type strain infection in mainland China (Cui et al., 2018).

Although a two-dose schedule of mumps-containing vaccines (MuCV) has been implemented since 2014, Shandong Province continues to report between 3,000 and 8,000 mumps cases annually. To assess the genetic stability of circulating mumps viruses following vaccine introduction, we compared mumps isolates from 2006–2007 (pre-vaccine) and 2018 (ten years post-introduction) based on whole-genome sequencing. At the sequence level, we performed three distinct comparisons. First, the overall nucleotide diversity across all 16 isolates combined was low (mean *p*-distances: 0.01–0.03 for most genes; Figure 3A). Second, the inter-group p-distances between the two temporal groups ranged from 0.005 to 0.016 at the nucleotide level and from 0.003 to 0.030 at the protein level. Third, we compared the two groups with respect to their respective sequence identities to the vaccine strain S79 (Figure 4). That is, for each group, we calculated the identity of each isolate relative to S79, and then compared these identity values between the two groups using Mann–Whitney U tests. Using Mann–Whitney U tests, we found no significant differences between the two groups for any of the seven structural proteins (BH-adjusted *p* > 0.05). The phylogenetic tree showed intermingling of the 2006–2007 and 2018 isolates; The degree of similarity between the strains circulating in Shandong in 2018 and those from 2006–2007 is at least not lower than the general degree of similarity observed among genotype F strains nationwide (Cui et al., 2017), all of these indicating limited genetic divergence between the two temporal groups at the genomic level. Importantly, the sequence identities of all seven structural proteins relative to the vaccine strain S79 did not differ significantly between the 2006–2007 and 2018 isolates, indicating that the genetic divergence between the field viruses and the vaccine strain remained largely constant over the ~12-year period, with no detectable progressive genetic drift. Structural modeling based on homology and computational mutagenesis further supports this view, revealing only minimal changes in backbone geometry between the field isolates and the vaccine strain, with RMSD values indicating a highly conserved overall fold. Regrettably, the moderate quality of the computationally predicted structural models (GMQE > 0.40, QMEANDisCo > 0.50) limits the precision of these interpretations; the observed RMSD values (0.055 Å and 0.035 Å) are well below the expected positional error for homology models of this quality range and should therefore be interpreted with caution. Also, it is important to note that these structural predictions are derived from computational modeling and do not assess protein stability, conformational flexibility, receptor binding, antigenicity, or biological function; therefore, the observed conservation in backbone geometry should not be interpreted as definitive evidence for functional or antigenic preservation. Collectively, these findings suggest that no major antigenic divergence was detectable in the circulating viruses over the study period. Notably, the reduced genetic diversity of genotype F viruses in localized regions has been documented in China, suggesting regionally constrained viral evolution under immune pressure (Deng et al., 2012). Therefore, in addition to genomic surveillance of the virus, other contributing factors—such as waning vaccine-induced immunity, suboptimal vaccination coverage, or other host-related factors that have been observed in mumps outbreaks in other vaccinated populations (Gokhale et al., 2023; Rasheed et al., 2019; Ramanathan et al., 2018; Yao et al., 2025; Béraud et al., 2018; Donahue et al., 2020; Schenk et al., 2021; Yang et al., 2020)—warrant further attention.

Several key genetic variations were still observed compared to the vaccine strain. 2 sites under positive selection with a posterior probability exceeding 0.95 were found: site 34 (H) in SH protein and site 468 (F) in the NP protein. Recent studies have established the mumps virus SH protein as a pentameric viroporin with chloride channel function (Devantier et al., 2025). The C-terminal region of SH (approximately residues 35–57) forms an extended structure critical for pentamer stabilization (Devantier et al., 2025). Notably, site 34H is situated at the boundary of this functionally essential C-terminal segment, suggesting that substitutions at this position may affect SH stability, ion channel function, or host interactions—all linked to viral replication and immune evasion. For the NP protein, residue 468 lies near the C-terminus, a region whose functional role remains incompletely characterized (Zhang et al., 2021). Assessment of glycosylation sites and amino acid physicochemical properties identified one glycosylation loss (in MuVi/Linyi. CHN/26.18/1) across all seven structural proteins, along with eight mutations affecting hydrophobicity or charge in the F and HN proteins. These glycosylation and other key sites have all been reported in previous studies (Cui et al., 2013; Hüttl et al., 2020; Kyte and Doolittle, 1982; Waxham et al., 1987). These differences were also consistent with previous reports from Beijing, where all 38 circulating strains belonged to genotype F and differed from the vaccine strain at critical HN amino acid sites that determine cross-neutralization capacity (Chen et al., 2009). Besides, we observed 4–5% amino acid divergence in HN protein between Shangdong strains and vaccine strain S79. Although these amino acid substitutions may affect hydrophilicity or charge, their impact on antibody recognition remains uncertain. The F and HN proteins are the principal targets of neutralizing antibodies (Deng et al., 2012; Šantak et al., 2013), and mutations in antigenic sites could influence antibody binding (Shaikh et al., 2024; Gouma et al., 2016). However, neutralization assays using recombinant viruses carrying these substitutions are required to determine whether they affect immune recognition or represent neutral drift (Shaikh et al., 2024; Gouma et al., 2016). These observations suggest that future vaccine development efforts in China should consider genotype-matched vaccines targeting the predominant circulating F genotype to improve protection against mumps.

The *sh* gene region, known to be the most variable, is widely used for mumps virus genotyping (WHO, 2012; Gavilán et al., 2023). Our findings are consistent with this convention: phylogenetic analysis based on complete genomes largely agreed with the clustering obtained using the *sh* gene alone, with all Shandong strains grouping within genotype F in trees constructed from both datasets. However, discrepancies emerged in finer evolutionary lineage assignment. Strains MuVi/Jinan. China/12.06/1, MuVi/Binzhou. China/23.06, MuVi/Jinan. China/12.07, and MuVi/Zibo. China/11.06/2 exhibited inconsistent branching patterns at terminal nodes. Notably, MuVi/Zibo. China/11.06/2 clustered differently from reference sequences KF170916.1, MT350231.1, and KF170919.1 depending on the genomic region analyzed. Consistent with the established advantage of whole-genome over *sh*-gene for fine-scale phylogenetic resolution (Kidokoro et al., 2022), we employed whole-genome sequencing in this study, thereby facilitating comprehensive molecular surveillance of mumps virus.

Several limitations should be considered. First, the 16 genomes came from only two time windows (2006–2007 and 2018), precluding year-to-year evolutionary tracking. The modest sample size (*n* = 16) also limits the statistical power of selection analyses; thus, the two positively selected sites identified should be interpreted conservatively, and candidate sites (0.90 < posterior probability < 0.95) require validation in larger datasets. Second, the sample size is modest (*n* = 16), limiting statistical power. Third, structural modeling using SWISS-MODEL was performed only for sites meeting all of the following: posterior probability > 0.90, template coverage, and amino acid differences from the vaccine strain. This yielded two modeled sites—M20L in SH and I151V in F. However, the two sites with posterior probability > 0.95—SH 34H and NP 468F—lacked suitable template structures and therefore could not be modeled. Structural interpretation was based solely on backbone RMSD, without systematic assessment of side-chain conformations, solvent accessibility, or local electrostatic potentials. The moderate quality of the computational models further constrains the precision of our structural inferences. Fourth, we have not done functional tests to determine whether the identified mutations affect vaccine efficacy. Fifth, Neutralization assays or antigenic characterization were not performed in this study; therefore, we cannot formally exclude the possibility of undetected antigenic changes. In addition, our conclusions about vaccine effectiveness are limited—we compared sequences but did not measure neutralizing antibody titers or test viral escape *in vitro*. Sixth, our study focused on genomic characterization and did not integrate epidemiological variables—including individual vaccination history, population-level vaccine coverage, outbreak settings, or host immune status. We therefore cannot draw definitive conclusions, and further data are needed to evaluate the relative contributions of viral and host factors to persistent transmission. Finally, this study is regional. Within these limits, the data are hypothesis generating and provide a useful genomic reference.

## Conclusion

As the SH gene alone lacked sufficient diversity to resolve closely related isolates, whole-genome sequencing provided more accurate genetic characterization in this study. We provides a whole-genome-based, two-time point comparison of genotype F mumps viruses in Shandong across a ~ 12-year interval spanning vaccine introduction (2006–2007 vs. 2018). Comparative genomic analysis revealed phylogenetic intermingling of 2006–2007 and 2018 isolates, with low inter-group p-distances (<0.1) and no significant sequence differences in all seven structure proteins relative to the vaccine strain S79. These findings indicate that the genotype F mumps viruses circulating in Shandong over the ~12-year interval have remained genetically conserved at the sequence level with respect to the vaccine strain. However, we caution that these genomic observations alone cannot exclude the possibility of antigenic changes, and functional assays (e.g., neutralization tests) are required to assess potential impacts on vaccine effectiveness. Our results highlight the value of continued genomic surveillance to monitor viral evolution, while emphasizing that sequence-based inferences should be interpreted with appropriate caution.

## Data Availability

The sequences have been deposited in the GenBank database under accession numbers PV668532–PV668547.
